# DSCN: Double-target selection guided by CRISPR screening and network

**DOI:** 10.1371/journal.pcbi.1009421

**Published:** 2022-08-19

**Authors:** Enze Liu, Xue Wu, Lei Wang, Yang Huo, Huanmei Wu, Lang Li, Lijun Cheng

**Affiliations:** 1 Division of Hematology and Oncology, School of Medicine, Indiana University, Indianapolis, Indiana, United States of America; 2 Department of Biomedical Informatics, College of Medicine, The Ohio State University, Columbus, Ohio, United States of America; 3 School of Informatics and Computing, Indiana University, Indianapolis, Indiana, United States of America; 4 College of Public Health, Temple University, Philadelphia, Pennsylvania, United States of America; University at Buffalo - The State University of New York, UNITED STATES

## Abstract

Cancer is a complex disease with usually multiple disease mechanisms. Target combination is a better strategy than a single target in developing cancer therapies. However, target combinations are generally more difficult to be predicted. *C*urrent CRISPR-cas9 technology enables genome-wide screening for potential targets, but only a handful of genes have been screend as target combinations. Thus, an effective computational approach for selecting candidate target combinations is highly desirable. Selected target combinations also need to be translational between cell lines and cancer patients. We have therefore developed **DSCN (****d****ouble-target** **s****election guided by** **C****RISPR screening and** **n****etwork)**, a method that matches expression levels in patients and gene essentialities in cell lines through spectral-clustered protein-protein interaction (PPI) network. In DSCN, a sub-sampling approach is developed to model first-target knockdown and its impact on the PPI network, and it also facilitates the selection of a second target. Our analysis first demonstrated a high correlation of the DSCN sub-sampling-based gene knockdown model and its predicted differential gene expressions using observed gene expression in 22 pancreatic cell lines before and after MAP2K1 and MAP2K2 inhibition (*R*^2^ = 0.75). In DSCN algorithm, various scoring schemes were evaluated. The ‘diffusion-path’ method showed the most significant statistical power of differentialting known synthetic lethal (SL) versus non-SL gene pairs (*P* = 0.001) in pancreatic cancer. The superior performance of DSCN over existing network-based algorithms, such as OptiCon and VIPER, in the selection of target combinations is attributable to its ability to calculate combinations for any gene pairs, whereas other approaches focus on the combinations among optimized regulators in the network. DSCN’s computational speed is also at least ten times fast than that of other methods. Finally, in applying DSCN to predict target combinations and drug combinations for individual samples (DSCNi), DSCNi showed high correlation between target combinations predicted and real synergistic combinations (*P* = 1e-5) in pancreatic cell lines. In summary, DSCN is a highly effective computational method for the selection of target combinations.

## Author summary

Cancer therapies require targets to function. Compared to a single target, a target combination is a better strategy for developing cancer therapies. However, predicting target combination is more complicated than predicting a single target. Current CRISPR technology enables whole-genome screening of potential targets. But most of the experiments have been conducted on a single target (gene) level. To facilitate the discovery of novel target (combinations), we developed DSCN **(****d****ouble-target** **s****election guided by** **C****RISPR screening and** **n****etwork)** that utilize single target-level CRISPR screening data and expression profiles for predicting target combinations by connecting cell-line omics-data with tissue omics-data. DSCN showed great accuracy on different cancer types and superior performance compared to existing network-based prediction tools. We also introduced DSCNi derived from DSCN and designed specifically for predicting target combinations for single-tient patient. Our results showed synergistic target combinations predicted by DSCNi accurately reflected synergies on drug combination levels. Thus, DSCN and DSCNi have the potential to be further applied in the clinical personalized medicine practice.

## Introduction

The complexity of cancer is widely recognized, with heterogeneous disease mechanisms underlying primary, metastatic, and drug-resistant tumors [1,2]. Therefore, translational cancer research now focuses on the identification of combinational rather than single targets and the selection of drug combinations instead of single drugs [3,4]. The clustered regularly interspaced short palindrome repeats (CRISPR)-Cas9 knockout system is a revolutionary gene-editing tool. By the pooled CRISPR libraries, we can screen thousands of gene expression variation at one time. A CRISPR-based double knockout (CDKO) system has recently been developed to effectively screen gene pairs or target combinations by synthetic gRNAs (a short guide RNA) [5,6]. In this paper, we will use the terms gene pair and target combination interchangeably because they represent the same concept. However, screening using the CDKO system is limited by the number of genes to be screened. For instance, if we screen target combinations among 100 genes, and each gene has four gRNAs, there will be (4×100)^2^/2 = 80,000 combinations, a scale that is feasible in a CDKO system. However, across the genome, if we screen target combinations among 10,000 genes and select only one gRNA per gene, the resulting 10,000^2^/2 = 50,000,000 combinations will be practically infeasible. Therefore, a computational approach is needed to rank and select top candidate gene pairs from CDKO system.

There are two notable approaches in druggable target combination selection. OptiCon (optimal control nodes) [7] and VIPER (virtual inference of protein activity by enriched regulon analysis) [8]. Both approaches primarily utilize gene-expression data to construct a biological network, then rank and select druggable target combinations that demonstrate optimal control of the network. OptiCon takes a protein-protein interaction (PPI) network, a prior pathway knowledge, and multi-omics data (genomic and transcriptomic) as input. In OptiCon modelling, it used both signaling transduction and gene regulation information to rank and select these optimal control nodes (OCNs) as their combination targets among their networks. These top OCN pairs have the largest control of the network. VIPER [8], another method, relies on a pre-built mutual information network (i.e. gene regulartory network) using transcriptome data and ARACNE (Algorithm for the Reconstruction of Accurate Cellular Networks) information-theoretic algorithm [9]. VIPER infers a set of regulators, i.e. regulons, in a gene regulatory pathway. In VIPER data analysis, top ranked regulon pairs are selected based on the the number of their down stream regulated genes. In these two network based target combination selection algorithms, some top ranked control node pairs from OptiCon or regulon pairs from VIPER are shown to be synthetic lethal (SL) in validation experiments.

An SL gene pair refers to the loss of two genes that lead to cell death, but cell is still viable if losing one gene but not the other one. Network based target combination selection approach SL concept are technically different, but very much connected. Because some of the top ranked target combinations selected from the network were shown to be SL experimentally, they become SL discovery tools. In this paper, our proposed DSCN approach (i.e. Double-target Selection guided by CRISPR screening and Network) is indeed inspired by both network-based target combination selection approach and SL concept. Firstly, the spectral clustering and target selection scheme in DSCN is to select genes that have bigger impact on the network. Secondly, DSCN utilized CRISRP-Cas9 screening data in characterizing gene specific impact to cell viability. Then, taking advantage a novel subsequent sub-sampling scheme, DSCN is designed to select the first target that is highly essential in the network. In the subpopulation in which the first target is lowly expressed, the second target is selected based on its essentiality and network topology. In other words, the first target is selected for annihilating most of cells, and second target is selected is to annihilate the rest of the cells in which the first targets is lowly expressed (i.e. first target knockdown). Unlike VIPER and OptiCon, DSCN integrates SL concept into the target combination selection by a sequential selection for two targets.

VIPER and OptiCon did not address the translational connection between cell lines and tumor samples in selecting target combinations, but DSCN was designed to model this translation connection. Our ultimate goal is to select targets and/or target combinations for tumor tissues. Considering the potential difference between cell lines and tumor tissues, it is more important to identify important molecular subnetworks in tumor tissues than cell lines. Therefore, our DSC network and clustering analyses are performed on tumor tissue data first. Then, they are mapped to the cell lines for further target combination selection. On the other hand, to extend DSCN to predict target combinations for individual samples, a DSCNi tool is developed here.

## Materials and methods

### Datasets used in this study

**Table 1** lists these data sources used in paper, which include the types of cancer screened, data platforms and types, and sample numbers. We retrieved gene-expression and -mutation data for normal tissue and tumor samples for pancreatic and breast cancers from the Gene Expression Omnibus (GEO) [10,11] and The Cancer Genome Atlas (TCGA) [12] and gene-expression and -essentiality data from Project Achilles and DepMap [13–15], downloaded PPI data from STRING [16], extracted drug-target data from DrugBank [17], downloaded synthetic lethal gene-pair data from the SynlethDB database [18] and drug-sensitivity data from the DrugComb database [19].

**Table 1:** Datasets used in this study.

**Part 1. Multi-omics data**						
**Number**	**Cancer type**		**Data platform**		**Data type**		**Data (n, sample size)**
1	Pancreatic cancer cell lines		Affymetrix U133 2.0		Gene expression		GSE36133 (43), GSE46385 (7), GSE21654 (22), GSE17891 (20) Total sample size = 92
2			CRISPR screening		Gene essentiality		Project Achilles (v3.3.8) Total sample size = 26
3	Pancreatic tissue samples		Affymetrix U133 2.0		Gene expression (tumor)		GSE42952 (33), GSE51978 (2), GSE16515 (36), GSE15471 (39), GSE23952 (3) Total sample size = 113
4			Affymetrix U133 2.0		Gene expression (normal)		GSE46385 (3), GSE16515 (16), GSE15471 (39) Total sample size = 58
5			Illumina DNA-seq & RNA-seq		Mutation and gene expression (tumor)		TCGA ductal and lobular neoplasms (150), adenomas and adenocarcinomas (29)
6			Illumina RNA-seq		Gene expression (normal)		Solid tissue adjacent normal (41)
7	Breast cancer tissue samples		RNA-seq		Gene expression(tumor)		TCGA triple negative breast cancer sample (115)
8					Gene expression (normal)		TCGA triple negative breast cancer sample (163)
9	Breast cancer cell lines		Affymetrix U133 2.0		Gene expression		GSE36133 (12)
10			CRISPR Screening		Gene essentiality		Project Achilles (v3.3.8) Total sample size = 28
**Part 2. Databases**						
**Number**	**Data type**		**Database**		**Data**	
11	Protein-protein interaction (PPI) network		STRING [16]		PPI data in STRING database for human (v11): 11,609,230 interactions	
12	Drug targets		DrugBank [32]		Food and Drug Administration (FDA)-approved drugs and their associated target proteins: 1,769 gene targets,	
13	Synthetic lethal pairs		SynlethDB [17]		19,613 synthetic lethal gene pairs in human cancer	
14	Drug sensitivity data		DrugComb[18]		Drug synergies among cell lines on 5,226 drug pairs (HS578T)	

These types of data are organized as sets and utilized in the following ways:

GSE45757 is an independent set used for validating our proposed subsampling scheme. Set <1,2,3,4,11,12,13> is used as the training set for selecting the optimal scoring method, and the exploring set for the predicted impact of all target combinations from DSCN. (**Table 2**). Set <7,8,9,10,11,12,13> is used for external benchmark of predictions among DSCN and other methods. Set <1,2,5,6,11,12,13> is used as the exploring set for predicted impact of all target combinations from DSCNi (**Table 3**).

**Table 2:** Analysis of overall survival among the nine top-ranked target combinations in pancreatic ductal adenocarcinoma (PDAC). Here IS closes to the lower negative number is, the more support for synergy to the candidator of pairwise genes.

Gene 1	Gene 2	Impact Score (IS)	Log rank *P*-value	Hazard Ratio (HR)	HR *P*-value	Pathways
EGLN1	TFRC	-255.12	0.02	2.00	0.02	hypoxia, ferroptosis
MAP2K2	TFRC	-255.05	0.08	1.60	0.08	MAPK, ferroptosis
HPSE	TFRC	-255.01	0.19	1.50	0.20	Metabolism, ferroptosis
PPIC	TFRC	-254.86	0.06	1.80	0.06	Immune system, ferroptosis
FRK	TFRC	-254.86	0.04	1.80	0.05	Immune system, ferroptosis
EGLN1	COX7C	-254.79	0.84	1.10	0.85	Hypoxia, metabolism
XDH	TFRC	-254.75	0.001	2.40	0.002	Metabolism, ferroptosis
MAP2K2	COX7C	-254.72	0.14	0.65	0.15	MAPK, oxidative phosphorylation
FTL	TFRC	-254.71	0.10	1.60	0.10	ferroptosis, ferroptosis

**Table 3:** Contingency table between drug- and target-combination synergy.

Type	Predicted target-combination synergy	Predicted target-combination non-synergy
Drug-combination synergy	2,594	7,097
Drug-combination non-synergy	0	4,375

### Steps of DSCN algorithm

**Fig 1:**
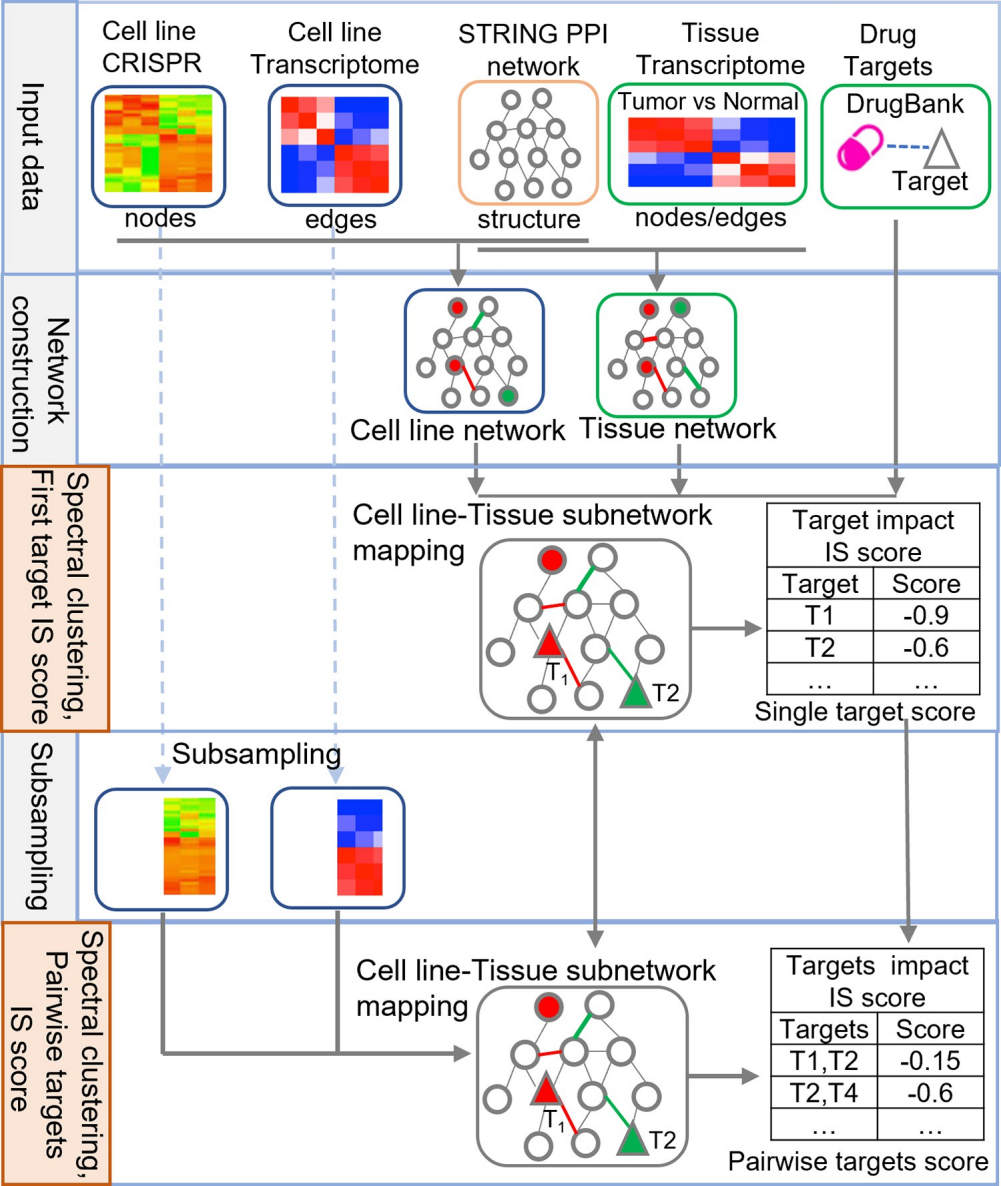
Overview of double-target selection guided by CRISPR screening and network (DSCN). There are five steps sequentially for pairewise targets identification in integrated networks of cell line and tissue. Step1. Input data includes cell line CRISPR gene knock-out data, cell line transcritome data, database STRING PPI network data, tissue transcriptom data and drug-target data from DrugBank. Step2. Perturbation network is constructed to cell line and tissue respectively by SCNrank [33]. Cell line network matches to tissue network and then seek the homology network module by spetral clustering in step 3. By SCNrank [33] target impact scores (IS) scoring, we will identify the first target in each spetral cluster. Then we sampling both of samples of cell line and tissue, and select those patients whose first targt gene with significant low expression in step 4. We will repeat steps2-3 to select the second target after the first target obtained in step 5, while the pairwise target gene IS score is calculated.

DSCN algorithm consists of six steps (**Fig 1**):

### Step 1: Network construction

In this step, we construct two integrated function networks, a tissue network *G*_*t*_ and a cell-line network *G*_*c*_. *G*_*t*_ consists of a skeleton from the STRING PPI network and edge weights from gene pair-wise Pearson correlations in tumor samples, and node weights are the fold changes in gene expression between tumors and normal tissue. A high fold change indicates higher gene expression in the tumor than in the normal tissue. Assume that there are a total of n genes (nodes) in *G*_*t*_. The affinity matrix *S*_*t*_ denotes the edge weights, and diagonal matrix *D*_*t*_ denotes the node weights in Eq (1):

$${G_{t}=S_{t}+D_{t},S_{t}}^{}=\left(\begin{array}{ccc} 0 & \cdots & w_{1n}\\ \vdots & \ddots & \vdots \\ w_{n1} & \cdots & 0 \end{array}\right),D_{t}=\left(\begin{array}{ccc} w_{1} & \cdots & 0\\ \vdots & \ddots & \vdots \\ 0 & \cdots & w_{n} \end{array}\right),$$ (1)

where *w*_ab_, *a*≠*b*∈(1, *n*) in *S*_*t*_ indicates the edge weight (correlation) between genes a and b in the tissue network; and *w*_*i*_ in *D*_*t*_ is the tumor versus normal fold change in the expression of gene *i*, *i* = 1,…,*n*.

Similarly, *G*_*c*_ consists of an identical skeleton from the same STRING PPI network and edge weights from pair-wise gene correlations in cell-line samples. Unlike *G*_*t*_, the node weight of *G*_*c*_ is from CRISPR-Cas9 screening data, which is indicated as the gene essentiality value. The gene essentiality value can be generally interpreted as the fold change in cell count before and after gene knockout. Genes demonstrating smaller fold change are more essential. In this study, all the essentiality values are log2 transformed. Similarly, *G*_*c*_ is decomposed into affinity matrix *S*_*c*_ for edge weight and diagonal matrix *D*_*c*_ for node weight in the cell-line network *G*_*c*_ = *S*_*c*_+*D*_*c*_.

### Step 2: Construction of Laplacian matrices for the tissue and cell-line networks

A Laplacian matrix measures all properties of a network, including node weight, edge weight, and connectivity. In this second step, we construct Laplacian matrices for the tissue network *G*_*t*_ and the cell-line network *G*_*c*_ as:

$$L^{}=D-S,$$ (2)

in which *D* is the diagonal matrix and *S*, the affinity matrix, defined in Eq (1), and *L*_*t*_ is the Laplacian matrix for the tissue network and *L*_*c*_, that for the cell-line network.

### Step 3: Spectral clustering for tissue network

We perform spectral clustering only on the Laplacian matrix of the tissue network *L*_*t*_ as:

- Normalize the Laplacian matrix *L*_*t*_ to *L*′_*t*_:
$${L\prime }_{t}=\left(\begin{array}{ccc} w_{1} & \cdots & -\frac{{abs(w}_{1n}w_{1})}{\sum_{k=1}^{n}{abs(w}_{1k})}\\ \vdots & \ddots & \vdots \\ -\frac{{abs(w}_{n1}w_{1})}{\sum_{k=1}^{n}{abs(w}_{nk})} & \cdots & w_{n} \end{array}\right)$$ (3)
  In the normalized Laplacian matrix *L*′_*t*_, all diagonal elements are positive, and all other elements are negative. The row sum of non-diagonal elements is equal to its corresponding diagonal. *abs* is absolute value.
- Perform eigen decomposition for matrix *L*′ to obtain the spectrum *E* = {*λ*_1_, *λ*_2_…*λ*_*n*_}, where 0 = *λ*_1_≤*λ*_2_≤⋯≤*λ*_*n*_, and their corresponding eigenvector.
- Choose the *k* smallest non-negative eigenvalues {*λ*_*i*_,…,*λ*_*i*+*k*_} and their corresponding eigenvectors, and combine these *k* eigenvectors into an *n*×*k* matrix, *H*.
- In this *H* eigenvector matrix, each row represents a gene node, and *k* columns represent the coordinate values of a gene node. The row vectors in *H* are used to calculate the Euclidean distance between a pair of gene nodes. We then perform *K*-means clustering for *n* nodes. To select the number of clusters, *K’*, to produce a good fit, we calculate Hartigan’s number, which measures the quality of clustering results. We select the optimal *K’* and constrain it further to less than 10 for practical consideration. This spectral clustering leads to *K’* exclusive clusters (i.e., subnetworks). From the tissue network *G*_*t*_, subnetworks ${g_{t}}_{1},\ldots {g_{t}}_{K\prime }$ are classified.

### Step 4: Mapping the tissue/cell-line network and calculating the impact score of Target 1

The cell-line network *G*_*c*_ is then mapped to the spectral clusters, ${g_{t}}_{1},\ldots {g_{t}}_{K\prime }$, generated from tissue network *G*_*t*_ in Step 3. Because tissue network *G*_*t*_ and cell-line network *G*_*c*_ share the identical network structure, i.e., nodes and connections, *G*_*t*_ subnetworks, {${g_{t}}_{1},\ldots {g_{t}}_{K\prime }$} are mapped to *G*_*c*_ subnetworks $\left\{{g_{c}}_{1},\ldots {g_{c}}_{K\prime }\right\}$ using their common node names and connections.

**Fig 2:**
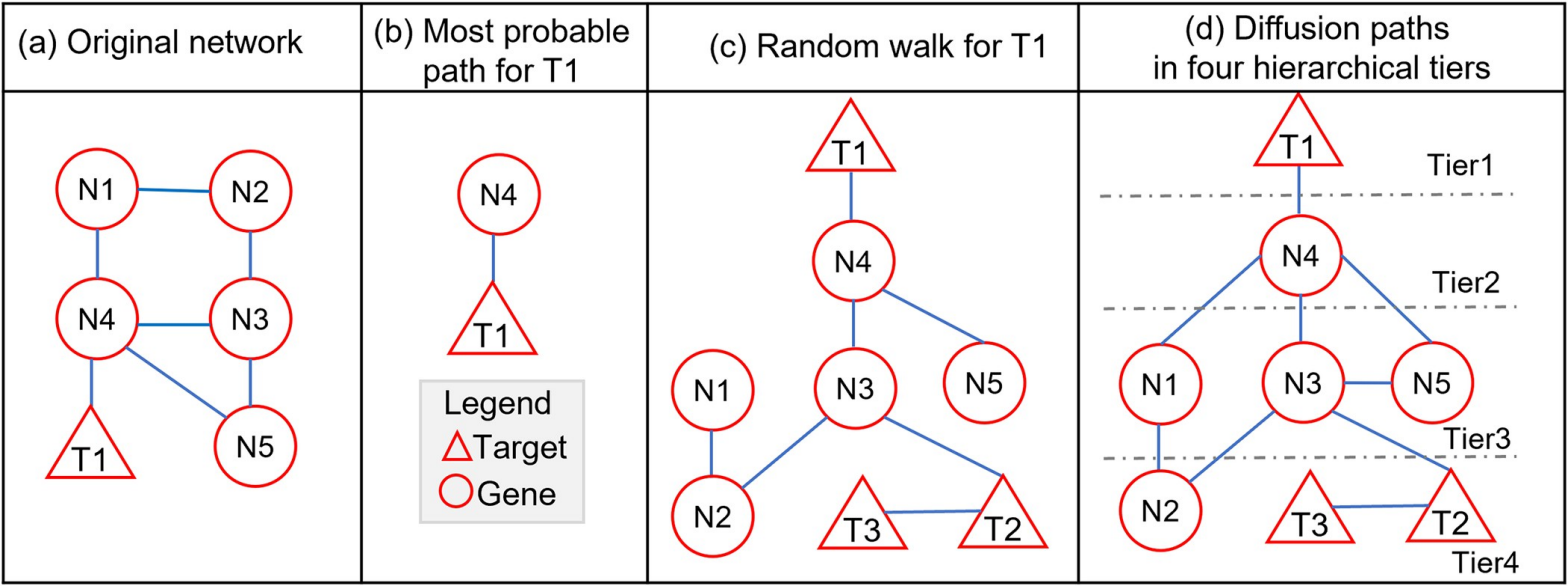
Network configurations for three methods “most probable path”, “random walk” and “diffusion path” are used to calculate target impact score (IS). (**a**) Original network. we use target T1 for example to denote the strategies in (**b**)-(**d**), (**b**) most probable path strategy. (**c**) random walk strategy. (**d**) diffusion path strategy by hierachical tier searching.

The target impact score will be calculated based on the cell-line subnetworks $\left\{{g_{c}}_{1},\ldots {g_{c}}_{K^{\prime }}\right\}$. We focus on all Food and Drug Administration (FDA)-approved drug targets (see **Table 1**) to calculate our target score. The impact score of a target 1 (*T*1) is calculated as the sum of the impact score itself and its impact on the rest of the genes in the network. Its general form is defined in Eq (4):

$$IS({T_{1}}_{})=S({T_{1}}_{})+\sum_{i\in \{1,..,n\}}S[N_{i}|Pa(N_{i})],$$ (4)

in which, {*N*_*i*_, *i* = 1,…*n*} are the gene nodes in the network other than *T*1, and *Pa*(*N*_*i*_) is a set of parent nodes of *N*_*i*_. In particular, the impact score on *N*_*i*_ depends on its parent nodes, *Pa*(*N*_*i*_). **Fig 2** illustrates the three different methods of calculating the impact score–the most-probable, random-walk, and diffusion paths.

#### Most-probable path

The immediate children of *T*1 are the gene nodes directly connected to *T*1, e.g., N4 is the direct child *T*1 in **Fig 2B**. In this method, we will count only the immediate children of *T*1 in calculating the impact score. Without loss of generality, let *ch*(*T*1) be the set of immediate children of *T*1. The most probable path of *T*1 is the one that has the smallest impact score among *ch*(*T*1). Based on the general impact score as calculated in Eq (4), the most-probable-path impact score is defined in Eq (5):

$$\begin{array}{l} IS(T1)=S(T1)+\min_{N_{i}\in \mathrm{ch}(T1)}S[N_{i}|T1]\\ =w_{T1}+\min_{N_{i}\in \mathrm{ch}(T1)}\left(w_{N_{i}}\times w_{T1,N_{i}}\right), \end{array}$$ (5)

where $w_{T1}\;and\;w_{N_{i}}$ indicate their node weights, and $w_{T1,N_{i}}$ indicates their edge weight.

#### Random walk path

The random-walk score is calculated in two steps. Step 1 is a random walk in the network, in which the random walk has a transition probability of traveling from one node to another. In **Fig 2C**, starting from *T*1, each node *N*_*i*_ is randomly visited. Here we used normalized edge weight for transition probability as defined in Eq (6):

$$P_{j,i}=\frac{w_{j,i}}{\sum_{x\in e}w_{j,x}},$$ (6)

where *P*_*j*,*i*_ is the transition probability from *N*_*j*_ to *N*_*i*_, *w*_*j*,*i*_ is the edge weight between them, and ∑_*x*∈*e*_ *w*_*j*,*x*_ is the sum of all edge weights of *N*_*j*_. In this Markov process, a node can be visited multiple times. We set the total number of random-walk steps as 2*n*, where *n* is the total number of nodes in the network.

Then, in Step 2, we defined the parent node as the node that visited *N*_*i*_ first, i.e., *Pa*(*N*_*i*_). Hence, the impact score of *T*1 becomes:

$$\begin{array}{l} IS({T1}_{})=S({T1}_{})+\sum_{i\in \{1,..,n\}}S[N_{i}|Pa(N_{i})]\\ =S({T1}_{})+\sum_{i\in \{1,..,n\}}{w_{i}\times w}_{i,Pa(N_{i})}. \end{array}$$ (7)

#### Diffusion path

Starting from *T*1, each node is visited in a hierarchical order. Therefore, the parent nodes of a node, *N*_*i*_, can be from the upper tier, i.e., *UpperTier* (*N*_*i*_), or the same tier, i.e., *SameTier* (*N*_*i*_). For instance, in **Fig 2D**, there are four tiers in the hierarchical structure starting from *T*1. The impact of *T*1 transmits from Tier 1 to Tier 4 in the network. Therefore, the impact score is defined in Eq (6):

$$\begin{array}{l} IS({T1}_{})=S({T1}_{})+\sum_{i\in \{1,..,n\}}S[N_{i}|Pa(N_{i})]\\ =S({T1}_{})+\sum_{i\in \{1,..,n\}}\left\{\sum_{j\in UpperTier}W_{ij}W_{i}+\sum_{w\in SameTier}W_{iw}W_{i}\right\} \end{array}$$ (8)

These three scoring methods are selected because of the following reasons. Firstly, for a undirected network, the distance between two nodes is defined as their Dijkstra shortest distance, which is equivalent to the most probable path in our case [20]. Secondly, a weighted and undirected network is also called ‘Markov Random Field’ [21], where Markov property[22] exists among all nodes. Random Walk based algorithms are frequently used in Markov random field [23,24], to mimic the traverse under Markov property: the current step only depends on the previous step. Thirdly, diffusion method is rather a deterministic approach, in which the impact of the target is weighted by the correlations among neighboring nodes and gene essentiality score of the nodes. Starting from the target node, the hierarchical structure of node tiers is determined from the topology of the network.

### Step 5: Subsampling and Target 2 (*T2*) score and selection

Once *T1* is selected, we remove cancer cell lines with higher expression of the *T1* than its sample mean and only keep cell lines with its expression lower than mean.This subsampling method characterizes the knockdown of the *T1*. Similarly, we also remove cancer cell lines with higher *T1* essentiality scores than the sample in our subsampling. After the resampling, we construct the cell-line network *G*_*c*_ as Eq (2) using the subsampled cell-line subsamples. We follow the same **Step 3** in mapping *G*_*c*_ to {${g_{t}}_{1},\ldots {g_{t}}_{K\prime }$} and calculate the *T2* impact score following the same algorithms defined in **Step 4**. The *T2* impact score is then denoted as *IS* (*T*2|*T*1), because the subsampling and network depend on *T*1.

### Step 6: Calculation of impact score for target combinations

Because *T*1 and *T*2 and their impact scores are computed sequentially, the combinational impact score will consider both sequential orders in Eq (7), in which *T*1≠*T*2:

IS(T1,T2)=IS(T1)+IS(T2|T1) (9)

#### Tissue cell-line subnetwork similarity measure

We measure the similarity of each subnetwork pair <${g_{t}}_{i},{g_{c}}_{i}$>, *i*∈(1,…,*K*′) between tissue and cell-line using the following scheme:

- Normalization of node weight (diagonal)
  To make two subnetworks, ${g_{t}}_{i}$ and ${g_{c}}_{i}$, comparable, we normalize the cell-line diagonal matrix ${D_{c}}_{i}$ according to the tissue diagonal matrix ${D_{t}}_{i}$ using the following formula:
$${{D\prime }_{c}}_{i}=(\begin{array}{ccc} \frac{w_{c,i,1}\sum_{j=1}^{J}w_{t,i,j}}{\sum_{j=1}^{J}w_{c,i,j}} & \cdots & 0\\ \vdots & \ddots & \vdots \\ 0 & \cdots & \frac{w_{c,i,j}\sum_{j=1}^{J}w_{t,i,j}}{\sum_{j=1}^{J}w_{c,i,j}} \end{array}),$$ (10)
  in which *w*_*c*,*i*,*j*_ denotes the node weight *j*∈(1,*J*) in the cell-line subnetwork, and *w*_*t*,*i*,*j*_, that in the tissue subnetwork. *J* is the total number of nodes in ${g_{c}}_{i}\;and\;{g_{t}}_{i}$.
- Normalization of edge weight
  The Laplacian matrices for each subnetwork pair, <${g_{t}}_{i},{g_{c}}_{i}$>, *i*∈(1, *K*′), are defined similarly as Eq (3): ${{L_{t}}_{i}}^{}={D_{t}}_{i}-{S_{t}}_{i}$ and ${{L_{c}}_{i}}^{}={{D\prime }_{c}}_{i}-{S_{c}}_{i}$. After node-weight normalization, trace (${L_{c}}_{i}$) = trace (${L_{t}}_{i}$). Then, their edge weights (non-diagonal elements) are normalized accordingly using the formula:
$${L\prime \prime }_{}=(\begin{array}{ccc} w_{1} & \cdots & \frac{w_{1J}abs(w_{1})}{\sum_{j=1}^{J}{abs(w}_{1j})}\\ \vdots & \ddots & \vdots \\ \frac{w_{J1}abs(w_{1})}{\sum_{j=1}^{J}{abs(w}_{Jj})} & \cdots & w_{J} \end{array}).$$ (11)
  Until this step, all edges (non-diagonal elements) in both Laplacian matrices, ${{{L\prime \prime }_{t}}_{i}}^{}$ and ${{L\prime \prime }_{c}}_{i}$, acquired node features during normalization. We keep the original directions (positive or negative) of node weights and edge weights for the following distance calculation.
- Distance calculation
  For two corresponding subnetworks ${g_{t}}_{i}\;and\;{g_{c}}_{i}$ in tissue and cell-line, we calculate the distance using their normalized Laplacian matrices ${{{L\prime \prime }_{t}}_{i}}^{}$ and ${{L\prime \prime }_{c}}_{i}$:
$$Distance({g_{t}}_{i},{g_{c}}_{i})=\sum_{j=1}^{J}\sum_{l=1}^{J}{{{(L^{\prime \prime }}_{t}}_{i}}^{}(j,l)-{{L^{\prime \prime }}_{c}}_{i}(j,l)){}^{2},\;l\neq j,$$ (12)
  where L′′(*i*,*j*) *i*≠*j* indicates the edge weight between nodes *l* and *j* in a given Laplacian matrix, and ${{{(L^{\prime \prime }}_{t}}_{i}}^{}(i,j)-{{{L^{\prime \prime }}_{c}}_{i}}^{}(i,j)){}^{2}$ indicate the Euclidean distance between the same edges in two Laplacian matrices.

#### Construction of a DSCN algorithm for an individual cancer cell-line sample (DSCNi)

We apply DSCNi algorithm for scoring target combinations in a single cancer cell line for a single patient. Very similar to DSCN, in building up *G*_*c*_, DSCNi relies on a set of expression profiles for a cancer cell line to calculate the edge weights (i.e., correlations) between gene nodes. However, unlike DSCN, DSCNi uses a cell-line-specific essentiality score for node weights. Its impact score calculation for *T1*, *IS*(*T*1), follows exactly from **Steps 1, 2, 3, and 4.** In modeling the knockdown of *T1* in the subsampling in **Step 5**, we maintain the same *T1* subsampling as DSCN, i.e., we remove samples with higher expression of T1 than its sample mean. However, we will keep the same essentiality score for this individual cancer cell-line sample to calculate the Target 2 impact score. We calculate the final combination target impact score similarly as in DSCN, such that it has a comparable meaning to that calculated from DSCN.

#### Analysis of association between drug- and target-combination synergy

The Bliss score [25] measures the synergistic effect of a drug combination, i.e., the effect of the drug combination on cell viability rather than the additive effects of its two component drugs. A two-drug combination is considered synergistic if its Bliss score exceeds 0.12 [26]. On the other hand, the target combination is predicted to be synergistic if the impact score of two target is smaller than the additive score of two individual targets, as in Eq (13), in which the impact scores of *IS*(*T*1, *T*2), *IS*(*T*1) and *IS*(*T*2) are calculated by (9) and (8). (Note: the impact score usually takes the negative value. The smaller, the more impactful).


$$IS({T1}_{},T2)<IS(T1)+IS(T2)$$ (13)

In this section, we will define an association analysis between drug-combination scores and target-combination synergy scores. Consider a cancer cell line screened by a set of drug combinations, and these drug combinations can be categorized as either synergistic or non-synergistic based on their Bliss scores. Then, for each drug combination, we identify all its two-target combinations, calculate their synergy scores, and classify the drug combinations as either synergistic or not as in Eq (13). In a 2 by 2 contingency table, the rows are drug synergy (Y/N), and columns are target synergy (Y/N). For each drug combination, all counts of target-combination synergy and non-synergy are added to the corresponding row with respect to drug-combination synergy or non-synergy. The association between drug- and target-combination synergy is tested using a *Chi-square* test.

## Results

### Validation of the subsampling scheme for determining the impact of target-gene knockdown in the DSCN algorithm

**Fig 3:**
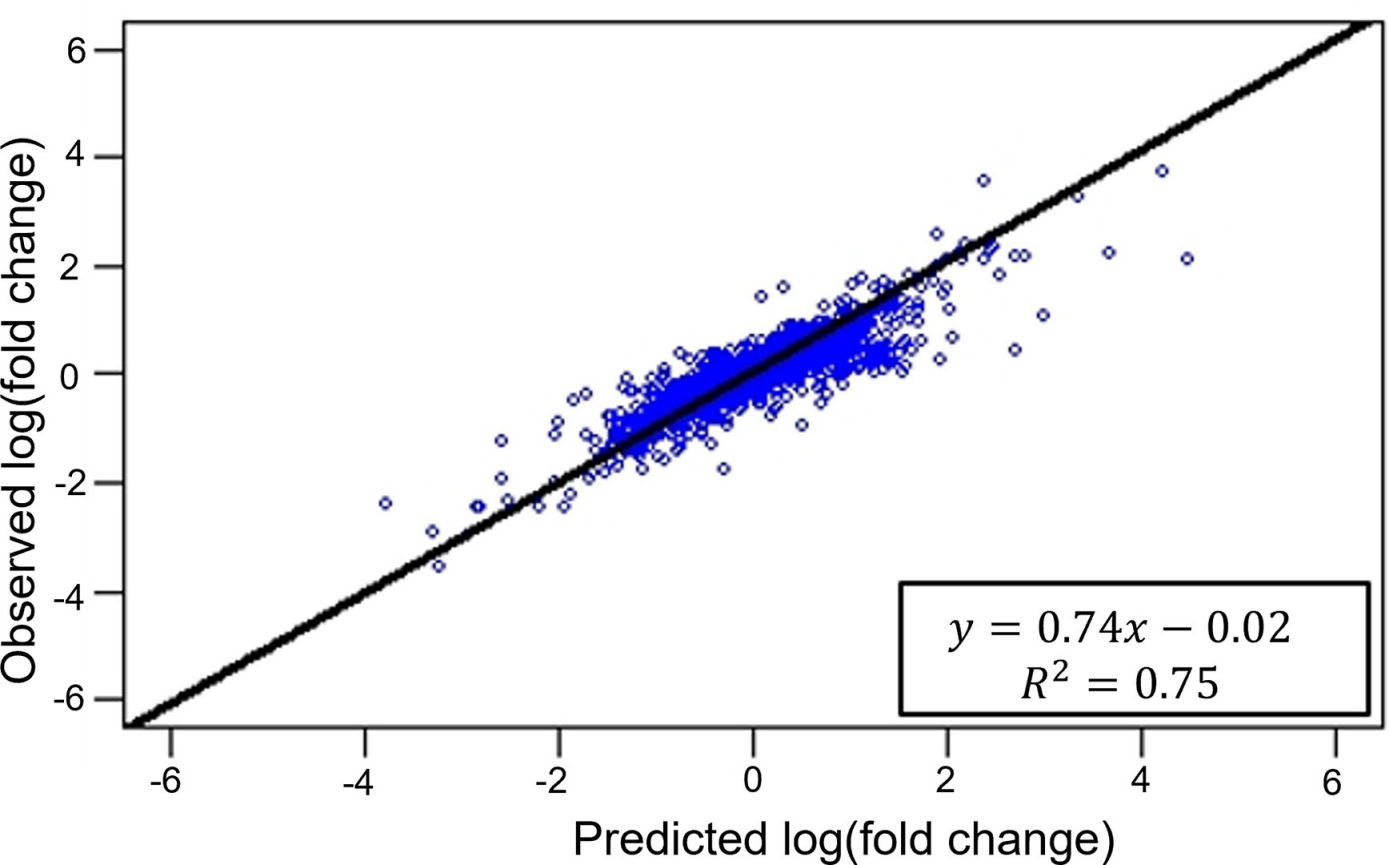
Correlation between the predicted and observed log-fold changes in gene expression among MAP2K1 and MAP2K2 neighbor genes in the protein-protein interaction (PPI) network.

In the DSCN algorithm, we designed our subsampling method (**Step 5**) to model the impact of Target 1 knockdown in the cancer cell line. To demonstrate the validity of this sampling scheme, we identified a GEO dataset, GSE45757, that provided transcriptome profiles across 22 pancreatic cell lines before and after MAP2K1 and MAP2K2 inhibition. Our analysis focused on 1,301 neighbor genes of MAP2K1 and MAP2K2 in the PPI network. Using the subsampling approach, we calculated the log-fold changes in these 1,301 genes between groups with either high or low expression of MAP2K1 and MAP2K2 group, which represent the predicted impact of Target 1 knockdown in the subsampling scheme. On the other hand, the observed log-fold changes in these 1,301 gene expressions were calculated during MAP2K1 and MAP2K2 inhibition. **Fig 3** shows a strong correlation, *R*^2^ = 0.75, between the predicted and observed fold changes among these 1,301 neighbor genes of MAP2K1 and MAP2K2. The findings of this analysis strongly support subsampling as a valid model for determining the impact of target-gene knockdown.

### Comparison of impact scores of target combinations using known synthetic lethal gene pairs in pancreatic cancers

**Fig 4:**
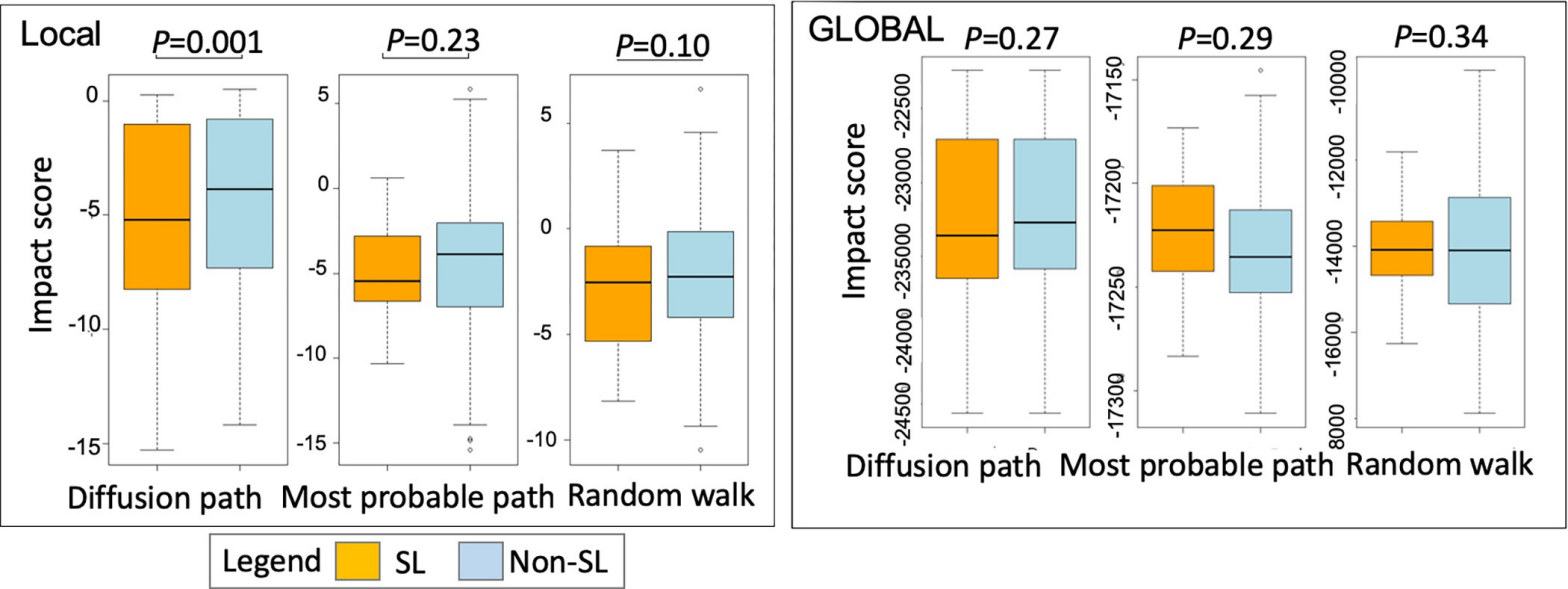
Comparison of target-combination impact scores using synthetic versus non-synthetic lethal gene pairs in pancreatic cancer. The three methods for calculating target impact score–the most-probable, random-walk, and diffusion paths are defined in **Fig 2**. The target impact scores (IS) are calculated from either the global protein-protein interaction (PPI) network (global) or the local PPI network (local).

We proposed three different scoring schemes to model the impact of target-gene knockdown on the network–those of the most probable, random-walk, and diffusion paths. In addition, the impact score can be calculated based on either the global or local PPI network (**Fig 4**). The local PPI network is the product of spectral clustering of the whole genome PPI network (global network). To compare the performance of these impact scores, we used the 23 reported synthetic lethal pancreatic gene pairs in SynlethDB as benchmarks. We compared impact scores between them and the other 164 gene pairs, which were derived from 21 unique genes among the 23 SL gene pairs. We constructed a tissue-function network using 153 tumor and 58 normal expression profiles of the pancreas from the GEO database (**Table 1**) and a cell-line function network using CRISPR screening data of 26 pancreatic cell lines from Project Achilles and 92 pancreatic tumor cell-line expression profiles from the GEO database (**Table 1**). All expression profiles are generated by Affymetrix U1332.0 microarray.

Smaller impact scores indicated the stronger impact of the gene knockdown on the network. Calculation of the impact scores using the local network generated from spectral clustering revealed a significant difference in diffusion-path-based impact scores (IS) between synthetic and non-synthetic lethal gene pairs (*P*-values) as well as lower impact scores of synthetic than non-synthetic lethal gene pairs. We observed the same trends with the other two impact scoring schemes, the most probable and random-walk paths, i.e., lower IS score in the synthetic than non-synthetic lethal gene pairs that were not statistically significant.

Calculation of the impact scores using the global network and diffusion-path scoring scheme also yielded lower diffusion impact scores in the synthetic than non-synthetic gene pairs, though the differences were not statistically significant. The scores of the most probable and random-walk paths, on the other hand, showed the reverse direction between synthetic and non-synthetic gene pairs. We, therefore, believe that using the diffusion path and local networks, evaluation of the target-combination impact score is an ideal approach in selecting synthetic lethal gene pairs (**Fig 4**).

### Compare the selection of target combinations among DSCN, OptiCon, and VIPER

We compared the performance of DSCN with that of two existing algorithms for the selection of target combinations–OptiCon and VIPER. Both of these use transcriptome profiles to select combination targets, and their top target combinations are master regulators of synergy that have optimal control of their corresponding networks. OptiCon requires tumor transcriptome profiles and corresponding mutation data as input to infer master regulators and predict synergies among them, whereas VIPER uses transcriptome profiles from both tumor and normal samples to select regulons and infers synergies among the regulons. Because the pancreas microarray expression profile used in the previous section has no corresponding mutation information, we utilized pancreatic expression profiles in TCGA to construct a tissue function network. We used 179 pancreatic tumor expression profiles along with their mutation data and 41 adjacent normal expression profiles (**Table 1**). We also used expression profiles of 92 pancreatic tumor cell lines from GEO and CRISPR-screening data of 26 pancreatic cell lines from Project Achilles (**Table 1**). Together, these data served for benchmark comparison of the performance of the three algorithms.

There are 14,066 overlapped genes (among tissue, cell-lines and STRING PPI network) as pancreatic cancer input in DSCN. Those genes create 14,066*14,065/2 gene pairs. Among these gene pairs, 37,275 are predicted to be SL in DSCN, i.e. their combination impact score is smaller than the sum of individual scores. There are 12,821 SL pairs within SynlethDB for all cancer types. Among them, only 79 SL pairs are pancreatic cancer specific. Among these 79 pairs, 23 correspond to FDA approved drug targets. SynlethDB evidence for these 79 pancreatic cancer SL gene pairs are based on experiments curated from literature, not from computational prediction. Hence, these 79 gene pairs are served as our bench marks in methods’ comparison.

In pancreatic cancer, DSCN predicted 37,275 synergistic target combinations, OptiCon, 2,778, and VIPER, 191. After mapping them onto 79 pancreatic cancer SL gene pairs, DSCN predicted 78 as SL. Hence the sensitivity is 78/79 = 0.99%. For 6,083 random combinations that were set as non-SL, DSCN predicts 5880 as negative. The specificity is 5880/6162 = 0.95. Of these 79, their predicted IS scores showed a 0.34 Spearman correlation with their SynlethDB score (*P* < 0.01), and the predicted IS scores were significantly lower than that of 6,162 random combinations on the t-test (*P* = 0.05). However, none of 79 pancreatic cancer SL gene pairs were predicted by OptiCon and VIPER.

These benchmark comparison analyses were performed on Indiana University’s supercomputer, ‘Carbonate’ [27]. DSCN completed its search of target combinations on the single central processing unit core in 12 hours, a significantly faster speed than those using OptiCon (320 hours) and VIPER (141 hours). Breakdown of major steps among three methods and their theoretical time complexities can be found in **S3 Fig**. DSCN completed its search of target combinations on the single central processing unit core in 12 hours, a significantly faster speed than those using OptiCon (320 hours) and VIPER (141 hours). This might be due to the time complexity of the three methods. In worst-case scenario, when the whole transcriptome network cannot be clustered into a subnetwork, the time complexity of DSCN can be described as O = (N3+2*(T2)(N2)(M2)), where *N* is the number of genes, *T* is the number of drug targets, and *M* is the number of samples. VIPER consists of two steps one is generating a mutual information network, which has a O = (*N*^3^+*N*^2^*M*^2^) time complexity. And there is no report on the time complexity of its second step. VIPER required permutation of 1,000 times of all samples to generate null model; thus we speculated that this might cause exceptionally high time complexity. OptiCon didn’t provide time complexity on three steps but judging from the source code, we speculated that Bayesian network models are applied on each subnetworks thus, searching the optimal structure would generate very high time complexity. The comparison results see **S1 Table.**

### Top-ranked target combinations and their associations with overall survival in patients with pancreatic cancer

We used expression profiles of tissues and cell lines from the GEO database (**Table 1**) to construct function networks and predict impact scores. Our dataset consisted of expression profiles of 153 tumors and 58 normal pancreas samples from GEO, CRISPR screening data of 26 pancreatic cell lines from Project Achilles, and 92 pancreatic tumor cell-line expression profiles from the GEO database. This yielded 14,066 overlapped genes.

In this analysis, we focused on 1,437 drug targets of all FDA-approved drugs in DrugBank and calculated their possible target combinations. Most interestingly, all genes in the top 230 target combinations are within the same subnetwork–the PDAC tissue subnetwork (**S1 Fig**) and cell-line subnetwork (**S2 Fig**). **S2 Table** includes the full list of genes in the subnetwork.

**Fig 5:**
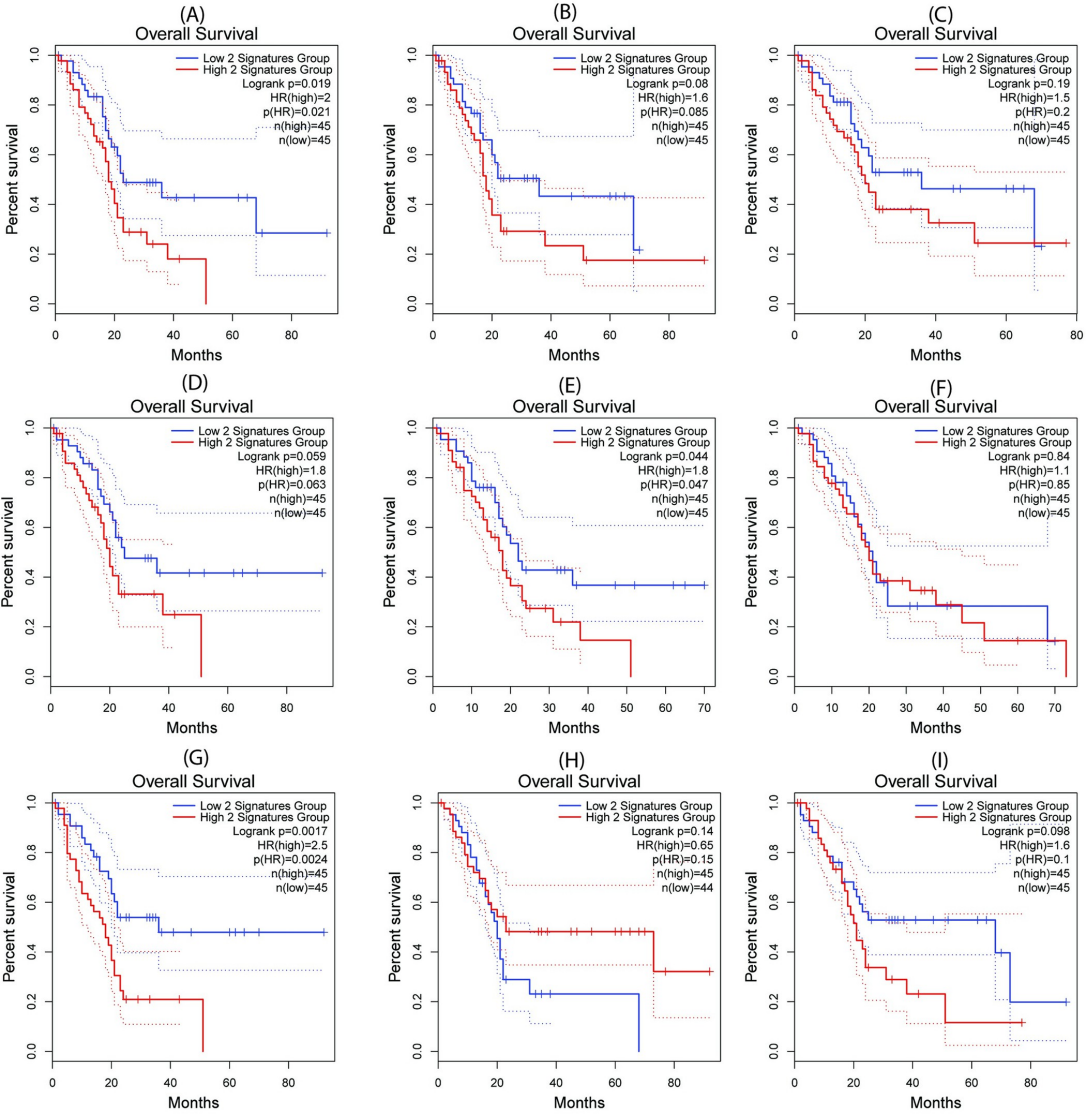
Kaplan-Meier curves for the nine top-ranked target combinations (a)-(i). Kaplan-Meier curves and other survival statistics for (a) < EGLN1, TRFC>, (b) < MAP2K2, TRFC>, (c) < HPSE, TRFC>, (d) < PPIC, TRFC>, (e) < FRK, TRFC>, (f) < EGLN1, COX7C>, (g) < XDH, TRFC>, (h) < MAP2K2, COX7C>, and (i) < FTL, TRFC>. Y-axis indicates survival probability while X-axis indicates months. The blue line in each plot indicates low expression of the two gene groups, and the red line, high expression.

**Table 2** displays the nine top-ranked target combinations and their annotations. Their Kaplan-Meier curves (**Fig 5**) are generated using TCGA PDAC clinical annotations from the Gene Expression Profiling Interactive Analysis (GEPIA) database [28]. Patient samples are categorized into two groups based on a target combination in which both genes are expressed either above (i.e., high-2) or below their means (i.e., low-2). Using log-rank test and Cox proportional hazard model to analyze the association between the expression of a target combination (high-2 versus low-2) and overall survival of patients with PDAC, we observed significant survival difference (*P* < 0.05, **Table 2**) of three of the nine top-ranked target combination comparisons, (EGLN1, TRFC), (FRK, TRFC), and (XDH, TRFC), their overall survival was worse for patients with high expression of these two genes than those with low expression.

Interestingly, seven of the top nine target combinations include transferrin receptor (TFRC), which encodes a surface receptor responsible for cellular iron intake. High expression of TFRC in PDAC and its strong association with PDAC growth and survival have been reported [29]. Recent studies suggest several key pathways of ferroptosis induction, including mitogen-activated protein kinases (MAPK) and reactive oxygen species (Ros) pathways [30]. Hence, targeting upstream genes (e.g., MAP2K2, EGLN2) along with downstream genes (e.g., TFRC, FTL) might lead to a synergistic effect.

### Performance of DSCNi in predicting drug synergy in cancer cell lines

DSCNi predicts target combinations for individual patients using gene-expression and -essentiality profiles. In this study, we assessed whether DSCNi predicted any association between target- and drug-combination synergy at each individual cell-line level. DrugComb [18] is a comprehensive database that incorporates information regarding the synergy of drug combinations from numerous well-known projects, such as the National Cancer Institute (NCI)-60 [31] for Human Tumor Cell Lines Screen. Because DrugComb includes only one PDAC cell line with five associated combinational drug treatments, we decided to use the cell-line data of triple-negative breast cancer (TNBC). We used 115 TNBC expression profiles from TCGA to generate edge weights in the tissue-function network, 12 TNBC cell lines from the Cancer Cell Line Encyclopedia (CCLE) database [32] to generate edge weights for the cell-line function network, and CRISPR screening data of the TNBC cell line “HS578T” from Project Achilles to generate node weights in the cell-line function network. Among all TNBC cell lines, HS578T has the largest number (N = 5,226) of drug-combination screening data in the DrugComb database, and our focus on drugs with known targets in DrugBank led to screening data for 1,031 drug combinations in the HS578T cell line. In turn, these drug combinations correspond with 14,066 target combinations in our network model (**S3 Table**).

To measure the association between predicted synthetic lethal pairs and synergistic drug combinations, we constructed a 2 by 2 contingency table (**Table 3**), in which rows correspond with drug-combination synergy (Y/N), and columns, with target-combination synergy (Y/N). Among synergistic drug combinations, synergy is predicted in 2,594 of their corresponding target combinations with DSCNi, but not in the other 7,097. Neither is synergy predicted in any of the other non-synergistic drug combinations in iDSCN. The *P*-value of the chi-squared test is 0.00001, and the odds ratio is 1,599. This is strong evidence of the greater likelihood that synergistic drug combinations have synergistic target combinations.

## Discussion

Our new DSCN method, double target selection guided by CRISPR screening and network, uses both cancer tissue and cell-line models to discover and rank target combinations, and it has several unique features and advantages in comparison with existing methods of selecting combination targets.

For the first time, DSCN uses a subsampling approach that characterizes the knockdown of the first target and models its impact on all the other genes. To demonstrate the validity of this assumption, we studied a set of transcriptome profiles across 22 pancreatic cell lines before and after MAP2K1 and MAP2K2 inhibition. Among 1,301 neighbor genes of MAP2K1 and MAP2K2 in the PPI network, our analysis revealed a high correlation of observed log-fold changes in these genes before and after MAP2K1 and MAP2K2 inhibition with log-fold changes calculated from the sub-sampling approach, *R*^2^ = 0.75.

DSCN also differs from all other methods by focusing on the overlapped functional network between cancer tissues and cell lines and further matching the differential gene expression in the tissue to gene essentialities in the cell line. This framework for the selection of target combinations is highly translational and practical. We investigated a number of scoring schemes for calculating impact scores, including the most-probable paths, random-walk paths, and diffusion paths, and we studied whether the global network and spectrum clustering-based local network lead to different calculations of impact scores. Using tumor samples of pancreatic cancer and cell-line samples and known synthetic lethal data in SynlethDB, we showed statistically significantly lower impact scores of target combinations in synthetic lethal gene pairs than other target pairs utilizing a diffusion-path approach on the local network. This analysis clearly demonstrates the validity of our proposed algorithm for calculating the impact scores of target combinations that reflect synthetic lethality.

Furthermore, DSCN is broadly defined for every target and target combination, unlike existing network-based target selection algorithms, such as OptiCon or VIPER, that are limited by their initial step in the selection of single targets (i.e., master regulators). This advantage of DSCN is demonstrated in the analysis of overlap among the top-ranked target pairs between DSCN, Opticon, and VIPER and synthetic lethal target pairs reported in the analysis of pancreatic cancer data in SynlethDB. DSCN identified 79 overlapped synthetic lethal target combinations, whereas OptiCon and VIPER showed zero overlaps. In addition, three of these top nine predicted synergistic target combinations in pancreatic cancer show statistically significant association with overall survival in patients with pancreatic cancer, and all three contain the TRFC gene, which encodes a surface receptor for cellular iron intake. Hence, the targeting of upstream genes (e.g., MAP2K2, EGLN2) along with downstream genes (e.g., FTL) might lead to a synergistic effect.

One caveat of our statistical association analysis between SL gene pairs and overall survival is its limited scope. We wanted to validate the SL gene pairs using clinical data, and attempt to correlate four combinations of high/low gene expressions between two genes with patient survival outcome. However, due to many high correlations among genes, small sample size quickly became a major problem when we created four groups of patients based on high/low gene expression between two genes. Consequently, we decide to compare one group that have low expression in both genes to the rest of the patients in overall survival. Although this comparison was not as ideal as a an SL validation, it at least indicates that at least knockout two genes have statistical and clinical significant effect on patient outcome.

SCNrank approach [33] is a single gene selection algorithm that we developed a couple of years ago. Both SCNrank and DSCN algorithms use the same types of omics-data as input, both algorithms do spectral clustering to a functional network; and both algorithms score the impact for target genes. However, DSCN generates the whole genome functional network. In DSCN, each gene can be either over-expressed or down-regulated in tumor versus normal expression. SCNrank, on the other hand, generated functional network that only contains nodes (genes) that are over-expressed. DSCN scores target 1 at first and scores target 2 given target 1 after. The sum of two scores will be the score for each combination. SCNrank only scores single target and do not have subsampling scheme.

In this paper, we investigated two relevant but different concepts, drug- and target-combination synergy, hypothesizing the greater likelihood of synergistic than non-synergistic drug combinations to target more synergistic target combinations. Using DSCNi, a model derived from DSCN for the prediction of target combinations for individual patients, we showed the truth of our hypothesis using triple-negative breast-cancer tissue and cell-line data. Based on 1,031 drug combination screening data in HS578T, a TNBC cell line, and its corresponding 14,067 DSCNi-predicted target combination synergy scores, we showed the 1,599-fold higher odds of synergistic than non-synergistic drug combinations to predict synergistic target combinations (*P* = 0.00001).

At the end, we state how our proposed DSCN and other network based target combination approaches can be utilized in cancer research. There is no doubt that these approaches can discover SL gene pairs. The SL concept itself has nothing to do with the normal cells or cancer cells. The application of SL to cancer research is to identify functional somatic mutations in a SL gene in cancer cell, while apply a drug to inhibit the other SL gene. This strategy would kill cancer cell, but not normal cells. DSCN approach will help us in identifying and validating these SL gene pairs in cell lines. Then, using patient genomics data, we shall further investigate whether one of the SL genes have functional mutations, while the one gene remains active. This will create an potential therapeutic drug target.

## Supporting information

S1 Fig Description: Subnetwork of TFRC from functional tissue network of PDAC. Dots and lines indicate genes and their interactions in protein-protein interaction network. Red dots: over-expressed genes in tumor versus normal samples. Blue dots: Down-regulated genes in tumor versus normal samples. Red lines: positive correlations between two genes on tumor tissue expression level. Blue lines: negative correlations between two genes on expression level.
(TIF)

S2 Fig Description: Subnetwork of TFRC from functional cell-line network of PDAC. Dots and lines indicate genes and their interactions in protein-protein interaction network. Red dots: genes with positive essentiality (knock-out result in reduced cell survival). Blue dots: genes with negative essentiality (knock-out result in increased cell survival). Red lines: positive correlations between two genes on tumor cell-line expression level. Blue lines: negative correlations between two genes on expression level.
(TIF)

S3 Fig Description: A demonstration of mapping tissue subnetworks to cell-line subnetworks. (TIF)

S1 Table Description: Breakdown of computational steps and their time complexities of three methods. (DOCX)

S2 Table Description: Subnetwork SL members in TFRB tissue (stable 2.1) and cell lines(stable 2.2). (XLSX)

S3 Table Description: Pairwise genes (matching drugs) with synthetic lethylity prediction Impact Score (IS) score in TCGA triple nettive braest cancer(TNBC) by DSCN algorithm calculation. IS score is compared with database DrugComb and SynlethDB real SL score data. Here, we includes 1437 drugs which all targets could cover.
(XLSX)
