# Supplementary figures and images for: DSCN: Double-target selection guided by CRISPR screening and network
Source: PLoS Comput Biol. 2022 Aug 19;18(8):e1009421. doi: 10.1371/journal.pcbi.1009421 (PMC9578612; doi:10.1371/journal.pcbi.1009421)

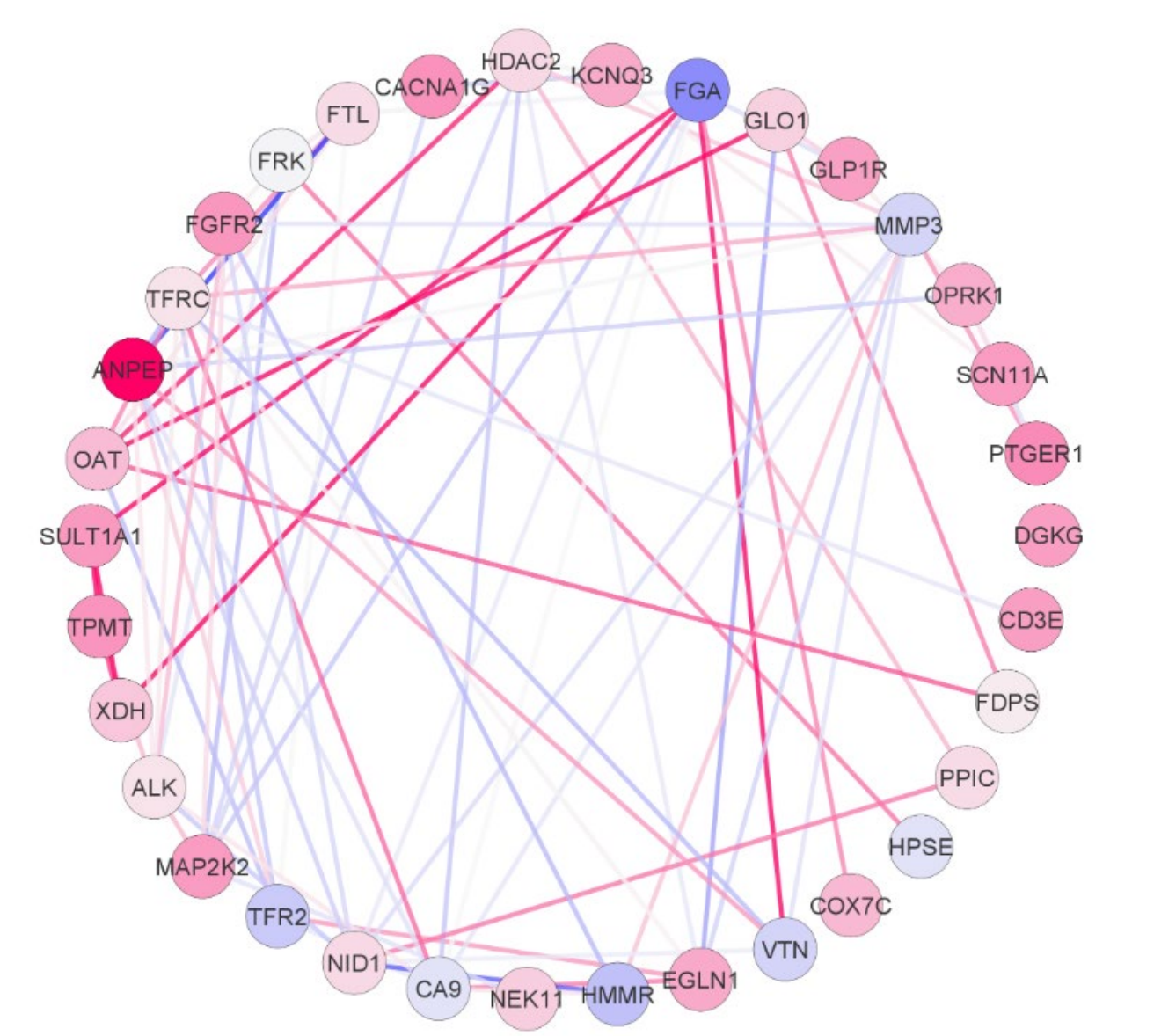

Supplement: S1 Fig — Dots and lines indicate genes and their interactions in protein-protein interaction network. Red dots: over-expressed genes in tumor versus normal samples. Blue dots: Down-regulated genes in tumor versus normal samples. Red lines: positive correlations between two genes on tumor tissue expression level. Blue lines: negative correlations between two genes on expression level. (TIF) [file pcbi.1009421.s001.tif]

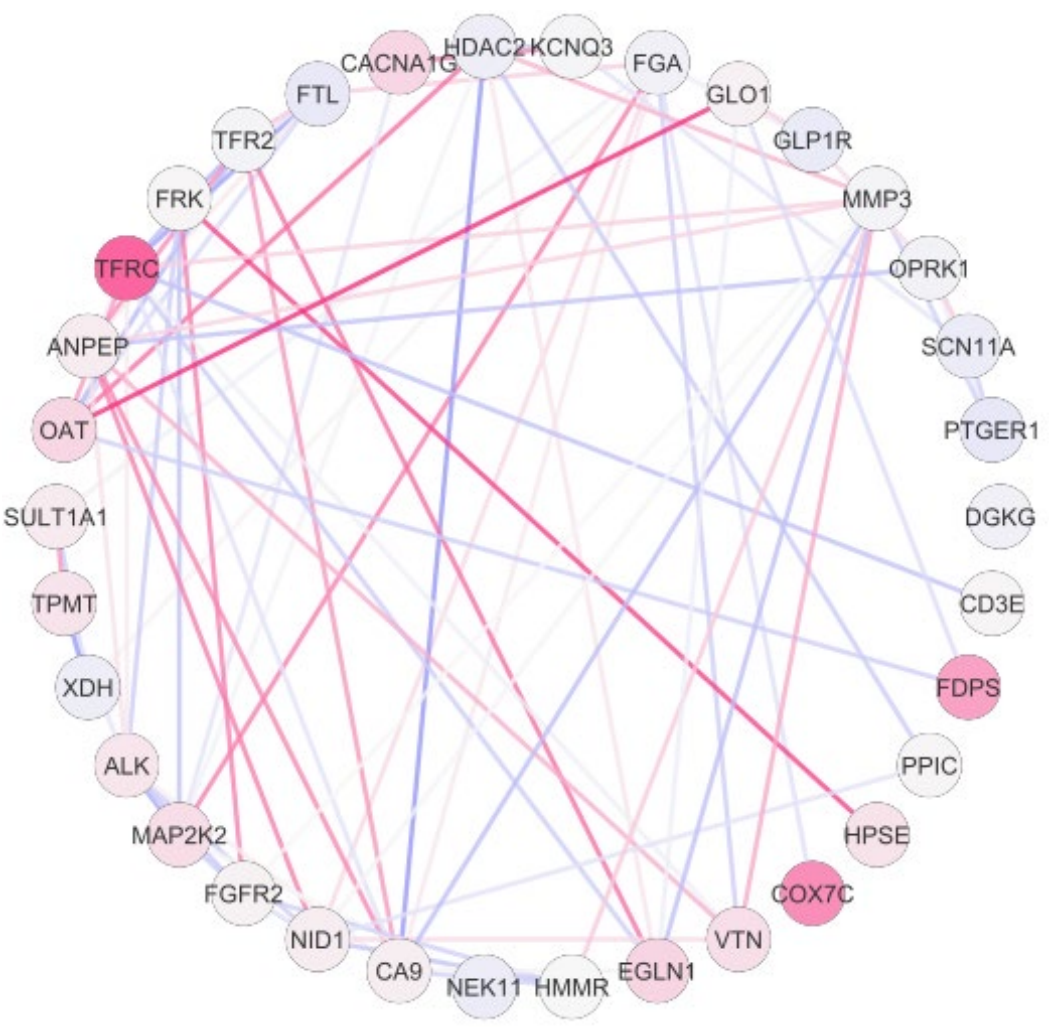

Supplement: S2 Fig — Dots and lines indicate genes and their interactions in protein-protein interaction network. Red dots: genes with positive essentiality (knock-out result in reduced cell survival). Blue dots: genes with negative essentiality (knock-out result in increased cell survival). Red lines: positive correlations between two genes on tumor cell-line expression level. Blue lines: negative correlations between two genes on expression level. (TIF) [file pcbi.1009421.s002.tif]

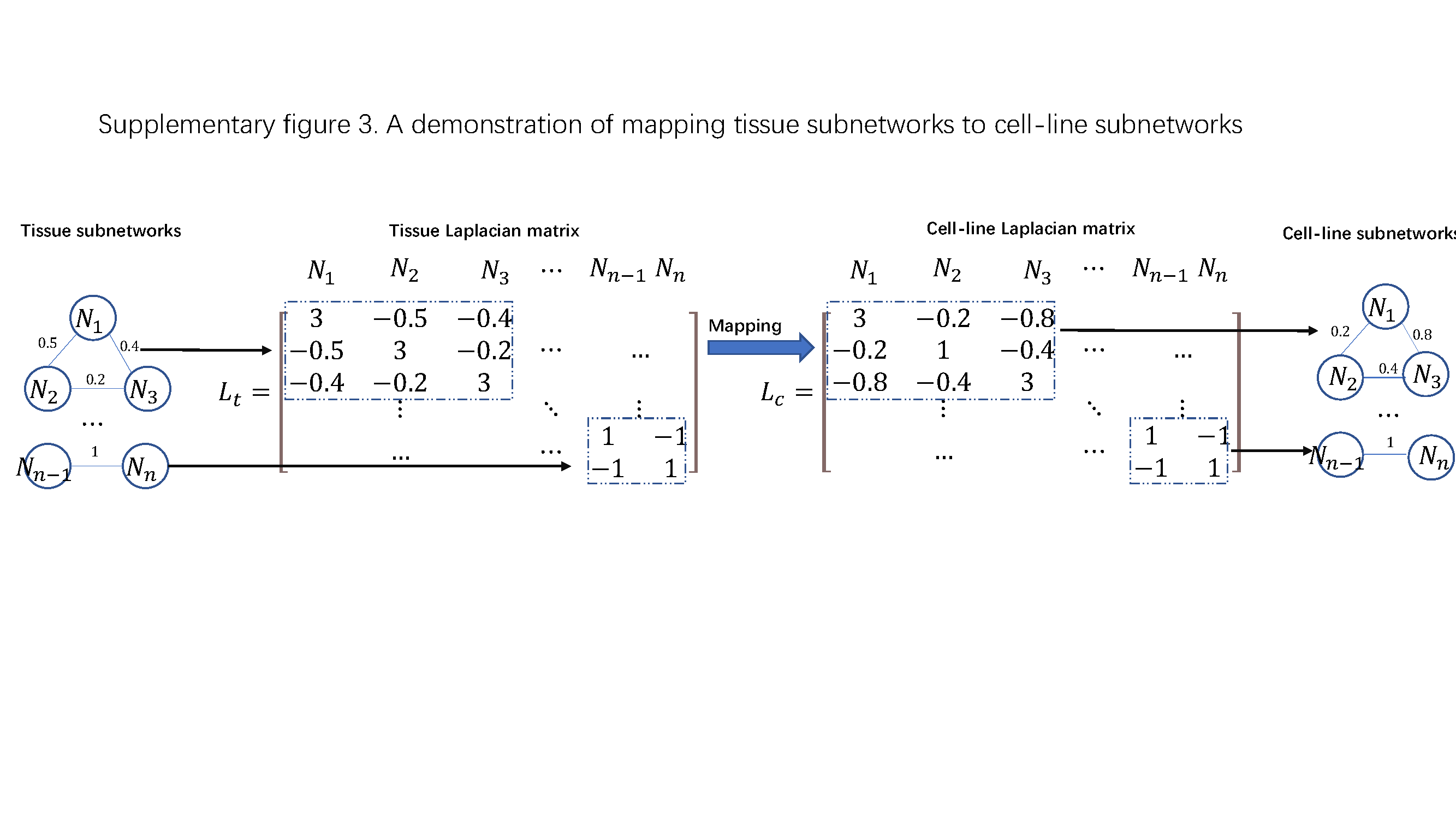

Supplement: S3 Fig — (TIF) [file pcbi.1009421.s003.tif]
