# Supplementary material for: DSCN: Double-target selection guided by CRISPR screening and network
Source: PLoS Comput Biol. 2022 Aug 19;18(8):e1009421. doi: 10.1371/journal.pcbi.1009421 (PMC9578612; doi:10.1371/journal.pcbi.1009421)
Supplement: S1 Table — (DOCX) [file pcbi.1009421.s004.docx]

**Supplementary table 1**. Breakdown of computational steps and their time complexities of three methods.

| Methods | Strategy | Network reconstruction  (NR) | NR time complexity | Target selection (TS) strategy | TS time complexity | TS actual runtime |
| --- | --- | --- | --- | --- | --- | --- |
| DSCN (our) | Graph transfer learning from cell line to tissue by CRISPR knock-out experiment guide, subsampling and searching. Searching strategies include "most probable path", "random walk”, and "diffusion paths" from whole network to subnetworks. | Homology perturbation networks of cell line and tumor by spectral clustering | $(N^{3})$ | Sequential scoring for the first target and pairwise targets | $(2\left( \begin{matrix} N \\ 2 \end{matrix} \right)\left( \begin{matrix} M \\ 2 \end{matrix} \right)\left( \begin{matrix} L \\ 2 \end{matrix} \right))$ | 12 *hrs* |
| VIPER^[2]^ | Gene regulator network reconstruction, and infer main regulators | Network reconstruction (ARACNe) | $(N^{3}+N^{2}M^{2})$ | Regulon inference | $NA$ | 141 *hrs* |
| OptiCon^[1]^ | Network reconstruction, selecting master regulators and synergy pairwise regulators | Null Hypothesis network reconstruction | Time complexity | Synergy evaluation | Time complexity | >320 *hrs* |

Note: $N$, $M,L$ are the number of the first, the second, and the third cycle in algorithm calculation
